# Simple and Tailorable Synthesis of Silver Nanoplates in Gram Quantities

**DOI:** 10.1021/acsomega.2c07452

**Published:** 2023-01-09

**Authors:** Rok Mravljak, Aleš Podgornik

**Affiliations:** † Department of Chemical Engineering and Technical Safety, Faculty of Chemistry and Chemical Technology, University of Ljubljana, LjubljanaSI-1000, Slovenia; ‡ COBIK, Mirce 21, 5270Ajdovščina, Slovenia

## Abstract

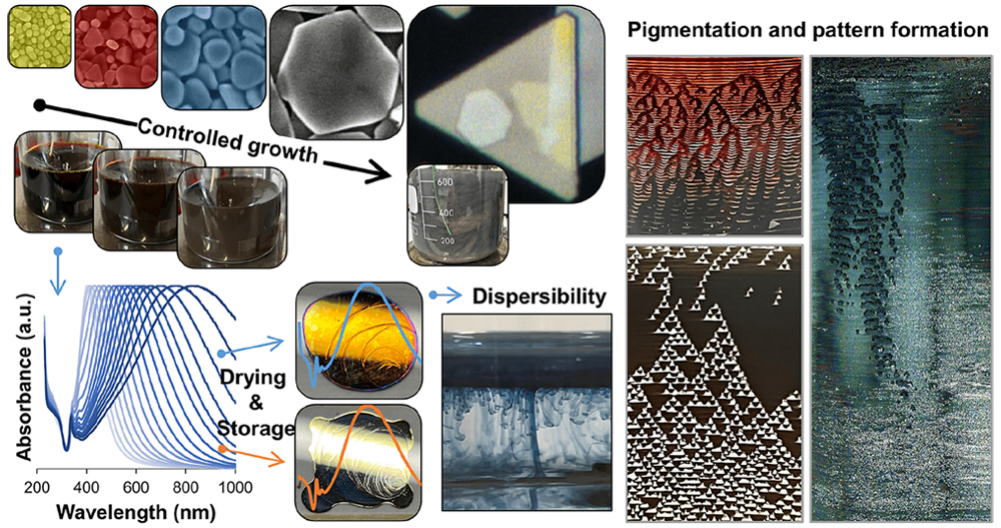

Due to plasmonic and catalytic properties, silver nanoplates are of significant interest; therefore, their simple preparation in gram quantities is required. Preferably, the method is seedless, consists of few reagents, enables preparation of silver nanoplates with desired optical properties in high concentration, is scalable, and allows their long-term storage. The developed method is based on silver nitrate, sodium borohydride, polyvinylpyrrolidone, and H_2_O_2_ as the main reagents, while antifoam A204 is implemented to achieve better product quality on a larger scale. The effect of each component was evaluated and optimized. Solution volumes from 3 to 450 mL and concentrations of silver nanoplates from 0.88 to 4.8 g/L were tested. Their size was tailored from 25 nm to 8 μm simply by H_2_O_2_ addition, covering the entire visible plasmon spectra and beyond. They can be dried and spontaneously dispersed after at least one month of storage in the dark without any change in plasmonic properties. Their potential use in modern art was demonstrated by drying silver colloids on different surfaces in the presence of reagents or purified, resulting in a variety of colors but, more importantly, patterns of varying complexity, from simple multi-coffee-rings structures to dendritic forms and complex multilevel Sierpiński triangle fractals.

## Introduction

Two-dimensional materials are of great interest to science because of their interesting physicochemical and applicable properties.^1,2^ Silver, which in bulk form has a silvery metallic luster in the absence of passivation, has been used since ancient times for art, trade, tools, and containers, and the number of applications is steadily increasing.^3^ In our time, scientists have discovered that metals, when very small, experience the so-called plasmonic effect caused by the collective motion of electrons via Coulomb interactions manifested in organized so-called “plasmon” oscillations.^4^ These interactions between light and a conductor are well-known for metal nanoparticles,^5^ patterned nanostructures,^6^ and 2D materials like graphene.^7^ Interestingly, the plasmon property of metal nanoparticles was exploited already as early as in Mayan^8^ and Roman^9^ times. It was found that the element silver has the smallest total plasmon oscillation damping rate and is therefore the best-performing metal in optical frequencies.^10^ Nowadays, there are a plethora of scientific publications on the controlled synthesis of silver nanoparticle colloids with different colors, depending on the size and morphology of the nanoparticles^11^ and the refractive index of the surrounding medium.^12^ One such morphology of silver nanoparticle colloids, that can be easily prepared in colors ranging from orange, red, purple, to blue, are thin silver nanoparticles in the form of plates with circular,^13^ triangular,^14^ and hexangular^15^ shapes. The cause of the nanoplate morphology is hexagonal close-packed (hcp) stacking faults along the length parallel to the {111} plane, which have a particularly low stacking-fault energy.^16^ The stacking faults are commonly observed by X-ray diffraction (XRD),^17^ transmission electron microscopy (TEM),^18,19^ and low-energy electron diffraction (LEED).^20^ It was found that the number and type of stacking faults determine the shape transformation into triangular and hexagonal nanoplates with mirror and center symmetry, respectively.^19^ Due to the pronounced plasmonic effect, silver metal nanoparticles have found applications exploiting their photothermal effects,^21^ for optoelectronic circuits,^21^ as light absorbers,^22^ for harvesting energy from microwaves to visible light,^23^ for improving cancer diagnosis,^24^ for their antibacterial activity,^25^ and for flexible electronics,^26^ colorimetric sensing,^27^ photoacoustic imaging,^28^ plasmon assisted chemistry,^29^ etc. In addition to the special optical properties of nanoplate morphology, it was found that stacking faults and twin boundaries at the edges of nanoplate crystals enhance the catalytic activity.^30^ This fact makes silver nanoplates more catalytically active^31^ compared to thermodynamically favored cuboctahedrons.^32^

Silver nanoplate morphology was first prepared in triangular form by a photoinduced method from synthesized silver nanospheres.^17^ Since then, many different synthesis methods have been developed, such as other light irradiation methods,^17,33−36^ biomimetic synthesis,^37^ and micelle directed synthesis,^13,18,38^ using ultrasound,^39^ at elevated temperatures,^40−45^ at room temperature or below,^15,46−56^ using a combination of room temperature, light, and heating,^57−59^ and on substrates.^60,61^ Despite a plethora of available methods, to our best knowledge, only two research groups have reported the preparation of highly concentrated silver nanoplate colloids.^15,52^ Both methods employ a multistep approach by first preparing a blue colloid of silver nanoplate seeds which consequently limited the smallest particle size that can be obtained to 140–150 nm.^15,52^ The first method employed a flammable solvent acetonitrile,^52^ while the second method requires the synthesis of silver thiocyanate, centrifugation, and vacuum drying as necessary intermediate steps.^15^ To produce plasmons which cover a broad range of the visible frequencies their size should also be smaller, since beyond crystals of certain size, colloids look typically silvery like bulk silver.^62^ It is therefore important to have a good control over a broad range of small particle sizes if the plasmon effect in the visible region is needed.

In this study we developed a simple, reproducible, controllable, and clean procedure to prepare silver nanoplates in gram quantities by extensive modification of the chemical synthesis protocol developed for the small scale synthesis of silver nanoplates in low concentrations.^57^ Furthermore, different final formulations for their storage, redispersion, and formation of surface patterns were investigated.

## Materials and Methods

Polyvinylpyrrolidone (PVP) with an average molecular mass of 50, 360, and 1000 kDa^63^ (PVP K30, PVP 360, PVP K90, respectively), silver nitrate (AgNO_3_), 96% denatured ethanol (EtOH), and antifoam 204 (A204) were purchased from Sigma-Aldrich, sodium borohydride (NaBH_4_) from Nokia Chemicals, and 30% hydrogen peroxide (H_2_O_2_) from Carlo Erba reagents. All chemicals were used as received without additional purification. Deionized (DI) water was used for all solutions.

### Silver Nanoplate Synthesis

The synthesis was developed by modifying previously published procedures.^31,56,57^ The typical synthesis proceeded by first preparing 18 mL of the growth solution with a final concentration of 7 g/L PVP K30 and 9.5 mM (1.61 g/L) AgNO_3_. The optimal amount of NaBH_4_ was determined by 24 consecutive additions of 100 μL freshly prepared 0.2 M NaBH_4_ in cold DI water to the solution while mixing. For UV–vis (Tecan, infinite M200Pro, Switzerland) measurements 10 μL samples were taken and diluted to 385 μL with DI water inside a 96-well polystyrene microplate, and DI water spectrum used as a blank was subtracted from all spectra.

Small spherical silver nanoparticles (AgNP) were prepared by adding 1 mL of freshly prepared 0.2 M NaBH_4_ in cold water to 18 mL of the growth solution. Transformation into silver nanoplates was initiated by slow consecutive additions of 40 μL of 30% H_2_O_2_.

The influence of PVP molecular mass was studied by testing PVP K30, PVP 360, and PVP K90 at the same mass concentrations of 7 g/L. The experiments to investigate minimal PVP to AgNO_3_ ratio were performed with PVP K30 in final concentrations of 4.28, 2.72, and 1.56 g/L.

The effect of foam formation on product quality was studied in two experiments using 90 mL of the growth solution to amplify the possible effect. In one experiment the solution was used as such, while in the second experiment an antifoaming polyether dispersion A204 was added. The pure viscous A204 and a 1 vol % A204 solution in 96% EtOH with much lower viscosity were used. Finally, 200 μL of 1 vol % A204 in 96% EtOH was found to be sufficient and was therefore used and added to the solution before the addition of NaBH_4_ in all further experiments, keeping the ratio with Ag^+^ constant. AgNP transformation was initiated by the precisely controlled addition of 30% H_2_O_2_ at a flow rate of 0.1 mL/min with a syringe pump (PHD 4400, Harvard apparatus, Holliston, MA, USA) via a polyether ether ketone (PEEK) capillary immersed into the growth solution.

Influence of the H_2_O_2_ addition rate on the nanoparticle transformation was further studied with the growth solution containing A204 antifoam by adding 2.2 mL 30% H_2_O_2_ at a flow rate of 0.1 or 100 mL/min with a syringe pump, mimicking slow and instant addition.

Scalability experiments in terms of high concentration were performed with a 10-fold higher concentration of PVP K30, AgNO_3_, and A204 in 3 mL of the growth solution. The amount of added fresh NaBH_4_ was either 1667 or 101 μL with a concentration of 0.2 or 3.3 M, respectively. Sampling volume of 1 μL was diluted to 385 μL with DI water for absorbance measurement.

Scalability experiments in terms of increased solution volume were performed with 3, 18, 90, and 450 mL of the growth solution, with appropriately adopted process parameters.

### Purification and Characterization of Silver Nanoplate Particles

The properties of colloids were evaluated by measuring their UV–vis absorbance spectra. Spectra were analyzed by changes in absolute absorbance, peak position, and peak full width at half-maximum (fwhm). Presented spectra were normalized by the localized surface plasmon resonance (LSPR) maximum.

Silver nanoplate purification was performed 24 h after synthesis to allow complete decomposition of residual NaBH_4_ and H_2_O_2_, by splitting the colloid into 50 mL centrifuge vials and centrifuging it (LACE16R, Colo lab Expert, Slovenia) for 100 min at 20 °C at 11140 G to induce particle settling. An orbital shaker (IKA KS 260, Germany) at 450 rpm was used to disperse the settled particles in 45 mL DI water. This process was repeated twice to remove most PVP and other potential impurities. The obtained samples were used for drying experiments and microscopy.

Drying was performed 24 h after synthesis at 60 °C in the dark for colloids with and without PVP by pouring the colloid in a polytetrafluoroethylene (PTFE) container. Dried colloids were dispersed and evaluated after one month in terms of UV/vis absorbance spectra and visual appearance. The same drying procedure was performed on glass slides and exposed to ambient light to stimulate potential secondary silver nanoplate growth.

Microscopy was performed by scanning electron microscopy (SEM) and optical microscopy. For SEM the sample carrier made of aluminum was first sanded, polished, and cleaned with EtOH, DI water, and ultrasound (ASonic, Slovenia). A 1 μL drop of the centrifuged concentrated colloidal solution was placed on the SEM carrier and allowed to dry at 60 °C. For optical microscopy a small drop of the colloid was placed on a clean glass slide and allowed to dry at 60 °C. Samples were examined with FE-SEM (Zeiss ULTRA plus, Oberkochen, Germany) and confocal laser scanning microscope (Zeiss LSM 700, Oberkochen, Germany).

### Deposition of Ag Nanoplates on Substrates

The prepared colloids were dried directly on glass substrates and varnished wood. More complex patterns were prepared on glass slides immersed in the colloids. The temperature and concentration of the particles and PVP were changed to control pattern formation.

## Results and Discussion

Several criteria were considered in the development of a scalable method for the synthesis of silver nanoplates. The method must be seedless, consisting of as few reagents as possible, enable preparation of silver nanoplates with desired optical properties in high concentrations, be scalable, and provide a final small volume formulation for storage and reuse. The thermal synthesis method^57^ comprised of AgNO_3_, NaBH_4_, H_2_O_2_, trisodium citrate, and PVP was used as a starting point. While trisodium citrate has long been considered essential for silver nanoplate formation, it was later demonstrated that it can be substituted by other carboxylic acids.^64^ During our preliminary investigation, we found that silver nanoplates can be produced even in the complete absence of trisodium citrate or other carboxylic acid^31^ and can already be obtained with only four reagents, namely, AgNO_3_, NaBH_4_, H_2_O_2_, and PVP. During further method development and optimization, the role of each component was carefully examined and optimized accordingly, also considering preparation in larger volumes and higher concentrations.

### NaBH_4_/AgNO_3_ Ratio Optimization

The first step in the synthesis of silver nanoplates was the preparation of small and stable silver nanoparticles (AgNP), which serve as a source of silver during the transformation. Various reducing agents such as H_2_(g), solvated alkali metal borohydrides, ascorbic acid, hydrazine hydrate, hydrazine dihydrochloride, and others are commonly used.^65^ Ultimately, NaBH_4_ was chosen because of its availability and reduction strength, which leads to a rapid nucleation and small AgNPs when properly stabilized.^66^ Since a borohydride anion has four H^–1^ atoms, it can donate a maximum of 8 electrons according to reaction^67^
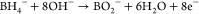
1or decompose in a spontaneous exothermic thermolysis or hydrolysis reaction influenced by various metal catalysts.^68^
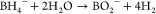
2

**Figure 1:**
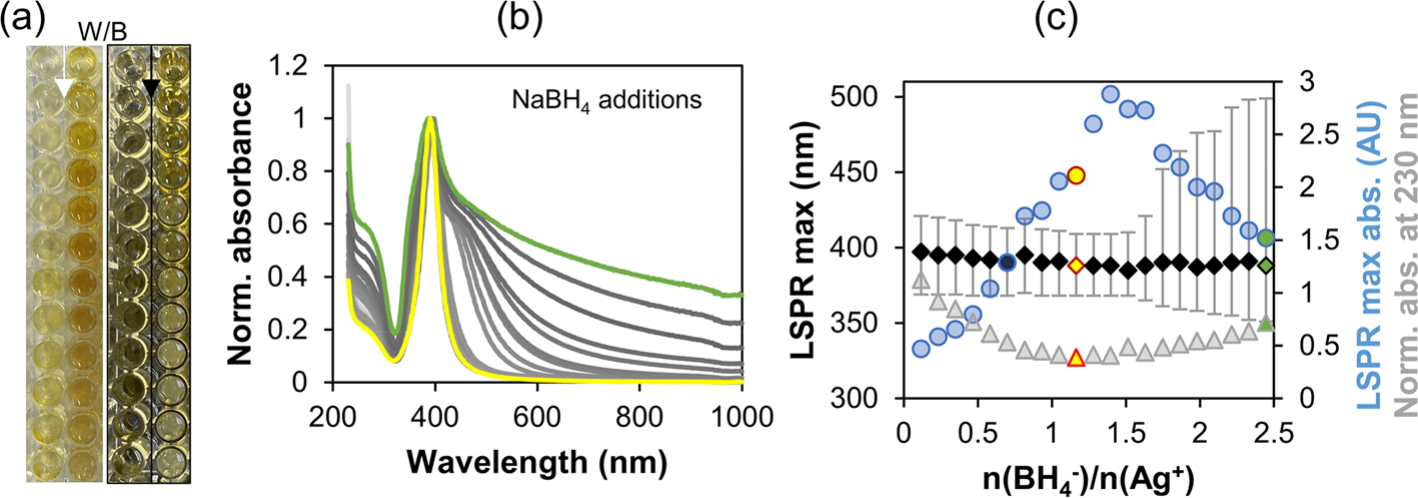
Optimization of the BH_4_^–^/Ag^+^ ratio. Photo of diluted AgNP samples on white (W) and black (B) background (a); UV–vis spectra after every addition of NaBH_4_ with the preferred spectrum plotted in yellow and the final spectrum in green indicating agglomeration (b); change in LSPR maximum position (black rhombi) together with the fwhm shown as error bars, the LSPR maximum absorbance (blue circles), the change in the normalized absorbance at 230 nm (gray triangles), and the optimal ratio (yellow points with red border), all as a function of the molar ratio of BH_4_^–^/Ag^+^ (c).

Both reactions leave borate salts as a byproduct. Therefore, it was necessary to find its smallest optimal amount. The results shown in Figure 1 indicate that much higher amounts than predicted according to reaction 1 are needed. This can be explained by the decomposition of BH_4_^–^ (reaction 2), which is confirmed by the formation of foam, and by the incomplete oxidation of BH_4_^–^, supported by the decreasing reactivity of the oxidation product.^67^ The designed experiment was performed by changing the molar ratio of BH_4_^–^/Ag^+^ by successive additions of equal amounts of BH_4_^–^. The diluted sampling solutions are shown in the photo in Figure 1a against a white and black background to better visualize their color and turbidity. Their normalized UV–vis spectra shown in Figure 1b were further analyzed as shown in Figure 1c. As presented in Figure 1c, the LSPR maximum increases steadily to a ratio of BH_4_^–^/Ag^+^ = 1.4, which was also reported in the literature.^56^ Afterward it decreases at almost the same rate due to nanoparticle agglomeration (data not shown),^69^ indicating that the ratio must be kept below this value. This result could already lead us to conclude that the ratio of 1.4 is optimal. However, we decided to analyze other parameters to see if even lower amounts of BH_4_^–^ can be used. A blue shift by 10 nm in the position of the LSPR maximum during BH_4_^–^ addition has stabilized beyond a BH_4_^–^/Ag^+^ ratio of 1.0 and the fwhm of the LSPR peak, indicated by the gray error bars in Figure 1c, decreased by 11 nm until the ratio of 1.17 and remained constant up to 1.4 while increasing dramatically thereafter. The normalized absorbance at 230 nm (gray triangles in Figure 1c), with an initial value of 1.12, decreases to a minimum of 0.39 at a BH_4_^–^/Ag^+^ ratio of 1.17 before increasing again. Since AgNO_3_ has the highest extinction (ε_230_ = 5.57 (g/L)^−1^cm^–1^) in the measured absorbance range at 230 nm, this suggests that the lowest concentration of free AgNO3 is reached. From the above observations, the optimum molar ratio of BH_4_^–^/Ag^+^ that gives the lowest fwhm, the lowest normalized absorbance at 230 nm, and the lowest NaBH_4_ consumption is at a value of 1.17. This ratio was therefore used in all further experiments.

### H_2_O_2_ Optimization

Once initial AgNP synthesis was optimized, we investigated AgNP transformation into silver nanoplates with a subsequent addition of hydrogen peroxide, keeping PVP/Ag^+^ ratio constant at 6.6. The ratio was chosen as an in-between value based on reports suggesting high ratio of 17.1^31^ and a low ratio of 0.89 and 0.22.^15^ H_2_O_2_ plays an essential role in nanoplate synthesis providing through its reaction energy for transformation of previously formed AgNP. A simplified set of reactions, excluding all possible intermediates for the reduction of Ag,^70^ can be presented as follows:
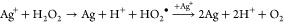
3

In the case of solid silver, the oxidation proceeds via corrosion^71^
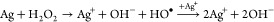
4
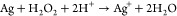
5or silver oxidation (Δ*G*° = −11.3 kJ/mol^72^) by the large amounts of oxygen formed at the silver surface and following Ag_2_O reduction by H_2_O_2_.
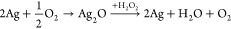
6

The consumption of H_2_O_2_ can occur also through the catalytic decomposition as follows:^73^
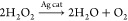
7

As seen from the above reactions, oxygen is produced by both the catalytic decomposition and, more importantly, the reduction of Ag^+^. This method lacks the carboxylic acids commonly used to protect Ag(111) facets,^64^ and oxygen is assumed to play the role of transforming AgNP into silver nanoplates by oxidative dissolution.^44^ In addition, molecular oxygen has been shown to chemisorb on Ag(111) facets even at lower pressures, forming disordered structures that gain sufficient mobility upon saturation at higher temperatures to nucleate into a new triangular superstructure phase.^74,75^

**Figure 2:**
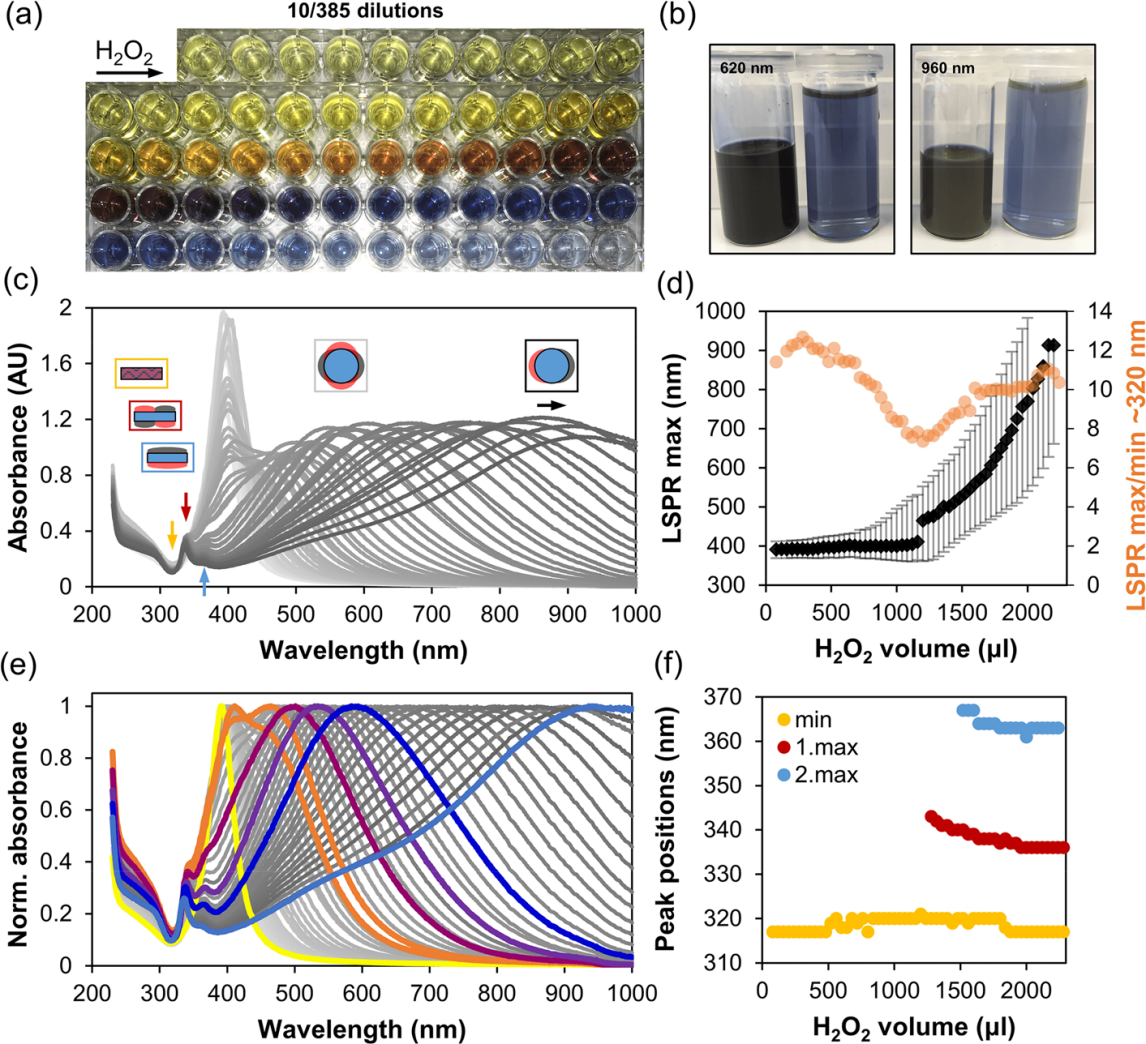
Synthesis of silver nanoplates in 18 mL of growth solution. Photo of diluted samples (a); photo of the two final colloids together with their 40-fold dilution (b); UV–vis absorbance spectra with arrows marking the analyzed peaks of the spectra together with schematics of the in-plane and out-of-plane resonance plasmons as well as the intraband transitions inside the metal (c); change in LSPR maximum position with the fwhm shown as error bars and the absorbance ratio of LSPR maximum to minimum at ∼320 nm (d); color marked normalized UV–vis absorbance spectra illustrating the color change of the colloid (e); peak positions (arrows in c) of minimum (yellow dots) and out-of-plane LSPR maximums (red and blue dots) (f).

The effect of H_2_O_2_ was investigated by consequently adding small amounts of 30% H_2_O_2_ into the growth solution. Addition was slow to allow foam dispersion between additions to minimize heterogeneity. Initially, no significant change in colloid color was observed, but once the transformation started it proceeded in a controlled manner, resulting in a variety of different colloids (Figure 2a,b). The absorption spectra shown in Figure 2c,e were then carefully analyzed, and four characteristic peaks shown in Figure 2d,f were traced. The intraband transition (yellow points in Figure 2f) inside the metal that damps the plasmon oscillations and leads to a minimum at ∼320 nm,^76^ the out-of-plane quadrupole at ∼335 nm and dipole plasmon resonance at ∼365 nm (red and blue points, respectively, in Figure 2f), as well as the most intensive in-plane dipole plasmon resonance at the LSPR maximum (black points with fwhm shown as gray error bars in Figure 2d).^77^ Another peak corresponding to the in-plane quadrupole plasmon resonance^76^ begins to appear beyond 500 nm for larger silver nanoplates. Spectra were also analyzed by calculating the LSPR maximum (Figure 2c, black arrow) and the minimum at ∼320 nm (Figure 2c, yellow arrow) absorbance ratio shown as orange points in Figure 2d. A ratio of LSPR maximum and the intraband transition damping was found to be a good indicator of the color intensity of the prepared colloid and can be used as quality control criteria during the synthesis of silver nanoplates.

### Effect of PVP Molecular Mass and the PVP/Ag^+^ Ratio

**Figure 3:**
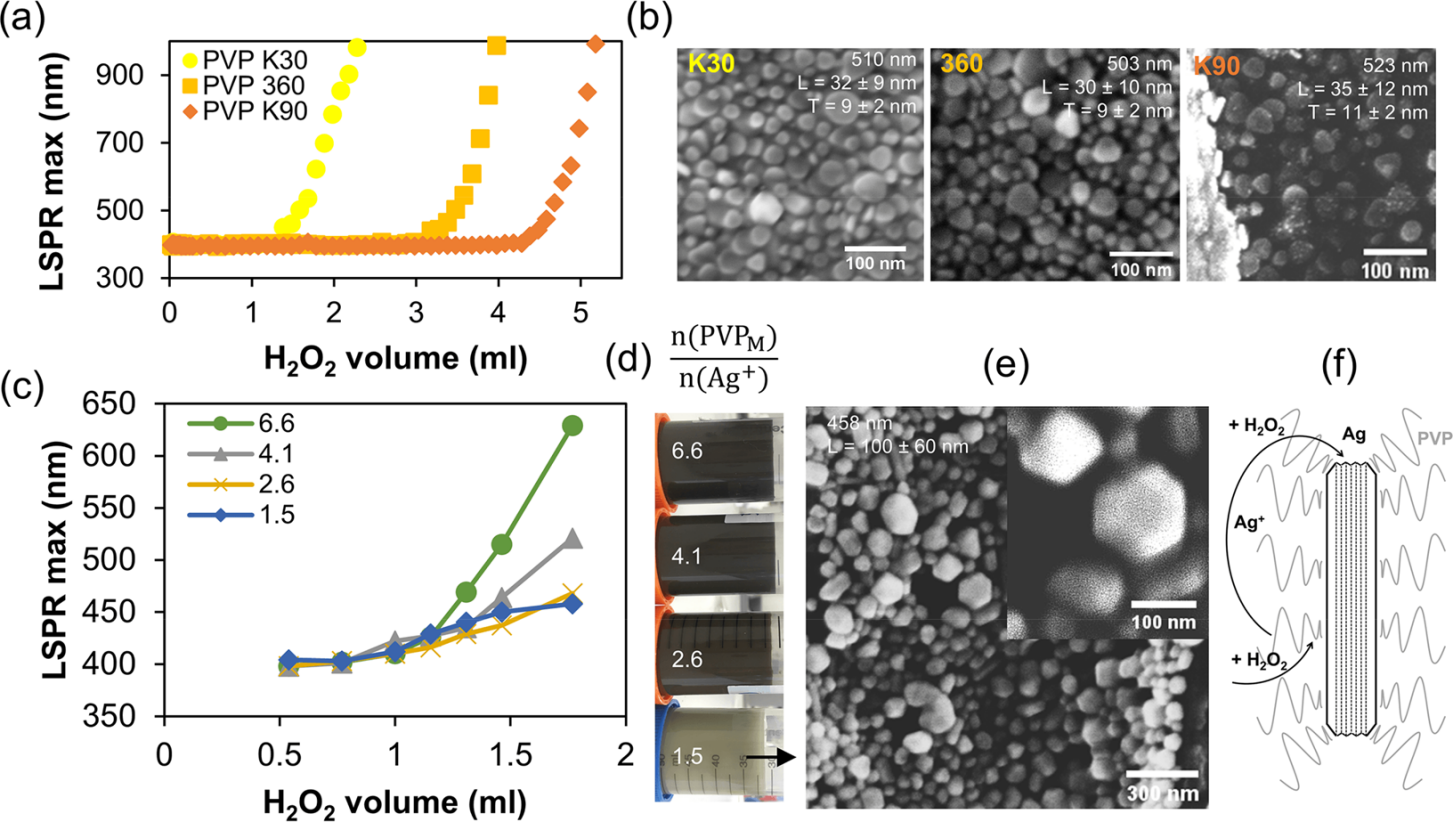
Effect of PVP molar mass and PVP_M_/Ag^+^ molar ratio of PVP K30 on silver nanoplate formation. Silver nanoplate formation during H_2_O_2_ addition monitored through LSPR maximum position for PVP polymers differing in molecular mass (a), with corresponding SEM images of formed silver nanoplates (b); silver nanoplate formation during H_2_O_2_ addition monitored through LSPR maximum position for different PVP_M_/Ag^+^ ratios (c), with the corresponding photos of the final colloids (d); SEM image of the colloid with the lowest PVP_M_/Ag^+^ ratio (e); schematic of an Ag nanoplate cross section with illustrated stacking faults, PVP steric coverage and the Ag ↔ Ag^+^ cycle (f).

Silver nanoplates have been prepared using a variety of nonionic and anionic polymers,^49^ but the cationic polymer PVP proved to be exceptionally good and was therefore preferred for the general synthesis of silver nanoparticle.^78^ PVP is mainly known for its stabilizing effect through surface adsorption and generated steric hindrance,^79^ its reducing tendency,^41^ its delocalized electrons between the pyrrolidone nitrogen and the carbonyl oxygen that allow efficient complexation of cations,^78^ and for being a good hydrogen-bond acceptor via the carbonyl oxygen.^79^ The results of the simulations showed that PVP binds more strongly to Ag(111) via van der Waals interactions, but direct chemical bonding is stronger on Ag(100),^80^ giving it the well-known facet capping properties for oriented crystal growth under certain conditions.^48^ Since PVP has been well studied under dilute conditions, common in nanoparticle synthesis, we expected the same properties at higher silver concentrations. It has been shown that the molecular mass of PVP has no effect on the thickness of silver nanoplates for one step reaction processes,^49^ but it has a significant effect on the thickness in multistep reactions.^48^ Therefore, we verified the effect of PVP molecular mass on formed nanoplates according to our procedure. In Figure 3b, the SEM images of silver nanoplates with an LSPR max at ∼500 nm, prepared with PVP K30, PVP 360, and PVP K90, show close similarity in their size and thickness, despite large differences in PVP molecular mass and in the amount of required H_2_O_2_ for their transformation (Figure 3a). The absence of thicker nanoplates confirms that the synthesis proceeds in one step^48^ and that the PVP molecular mass affects the transformation rate, indicating increasing steric stabilization with increasing polymer chain length, as expected.^79^ As steric repulsion increases, the number of successful collisions between AgNPs must decrease, and we hypothesize that this potentially affects the rate of transformation.

These data motivated us to further investigate the effect of PVP K30 since the lowest H_2_O_2_ amount was required for nanoplate synthesis. If we assume that each monomeric unit of N-vinylpyrrolidone (PVP_M_) attracts a cation, then *n*(PVP_M_) = *m*(PVP)/*M*(PVP_M_) = 9.00 mmol Ag^+^ can bind to 1 g of PVP, regardless of its molecular mass. A rather high PVP_M_/Ag^+^ ratio of 110 was originally used,^57^ which affects the solution viscosity^81^ and increases centrifugation time and, more importantly, PVP consumption. This ratio was already decreased recently;^31^ therefore, we investigated what can be the lowest ratio to obtain suitable nanoplates. In Figure 3c, colloids prepared with lower ratios resulted in a reduced transformation rate, and, even with an inflection point, not observed for higher ratios. Furthermore, the colloid with the lowest ratio looks comparatively milky (Figure 3d), and SEM images (Figure 3e) show the formation of large, thick crystals, which explains the loss of plasmon coloration. This demonstrates the importance of a stabilizing agent that allows unhindered formation of multiple stacking faults (Figure 3f), preventing growth out of the plane into large crystals. In summary, above the ratio of 1.5, a balance must be chosen between the benefits of lower H_2_O_2_ amount and lower viscosity, which ultimately depends on the application of the colloid produced. If silver nanoplates dispersed in water are required, the PVP concentration should be near the lowest ratio to allow fast centrifugation, while higher PVP concentration might be used when low H_2_O_2_ consumption is preferred.

### H_2_O_2_ Addition Rate

**Figure 4:**
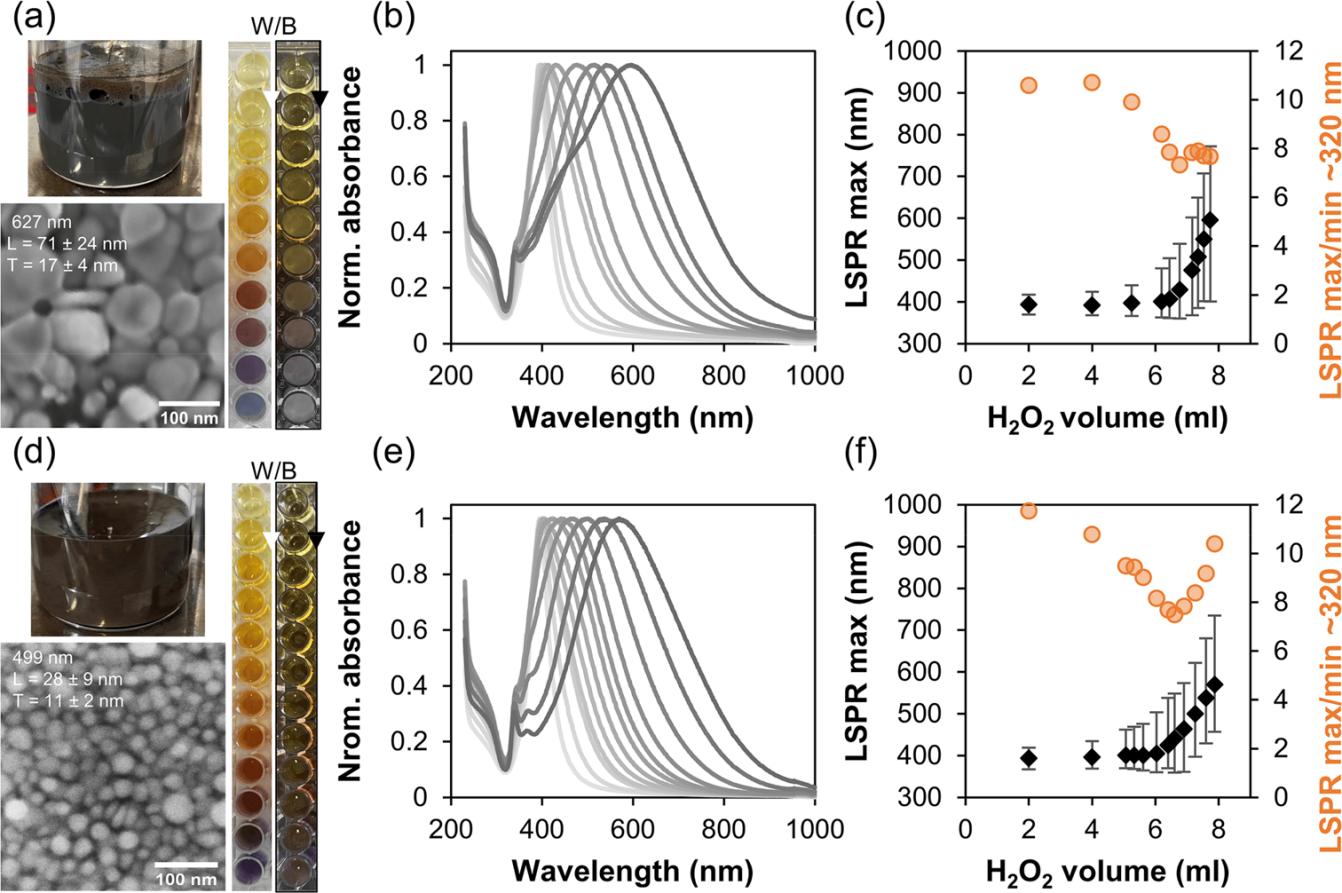
Effect of foaming investigated in 90 mL of growth solution (5 times the reference volume). Photo of 38.5-fold diluted samples over white and black background, photo of the final colloid and the corresponding SEM image for the synthesis without (a) and with (d) antifoam, corresponding normalized UV–vis absorbance spectra (b,e), change in LSPR maximum position with fwhm shown as error bars together with the absorbance ratio of LSPR maximum to minimum at ∼320 nm (c,f).

As mentioned earlier, foam formation was observed when BH_4_^–^ or H_2_O_2_ was added to the growth solution. The foam is rather stable, and it takes some time to disperse completely, which was found to affect the colloidal solution homogeneity and consequently silver nanoparticle polydispersity. To investigate the effect of H_2_O_2_ addition rate a syringe pump was used for precise control. Even when H_2_O_2_ was added at a slow flow rate of only 0.1 mL/min, this was not sufficiently slow to allow foam dissipation as shown in Figure 4a. This consequently led to broader peaks according to the measured fwhm and a lower peak max/min value shown in Figure 4c. Furthermore, additional experiments indicated that foam formation was minimized by further drastically reducing the H_2_O_2_ addition rate (data is not shown). Since this significantly prolongs preparation time and consequently process productivity, an alternative approach was tested. To reduce the foam formation, we used a water-insoluble and biocompatible polyether dispersion-based antifoam agent Antifoam A204. Experiments were performed with pure A204 as well as a solution of A204 in 96% EtOH to minimize its viscosity. Both provided the same results demonstrating that EtOH had no effect on the reaction; therefore, A204 dissolved in ethanol was further used for easier handling. At 0.222 per ten thousand, which was the lowest A204 concentration found to sufficiently prevent foaming, we observed that a stable A204/water emulsion formed above a temperature of 27 °C, which was noticeable by the formation of turbidity. Therefore, the temperature was monitored and always kept below this threshold during synthesis. Furthermore, to prevent H_2_O_2_ reaction with a silver layer floating on a top of the growth solution, a capillary was immersed in the solution to provide direct contact with the colloid. When comparing the same experiment without (Figure 4a-c) and with antifoam (Figure 4d-f), it can be noticed that foam formation affected the colloid resulting in a lower plasmon activity and slightly grayish coloration (seen by comparing photos in Figure 4a and d). This made us suggest that at the same addition rate of H_2_O_2_ thinner silver nanoplates might be obtained when an antifoam agent is used to minimize foaming. The hypothesis was later confirmed by SEM observations showing that on average slightly thicker crystals formed without antifoam.

**Figure 5:**
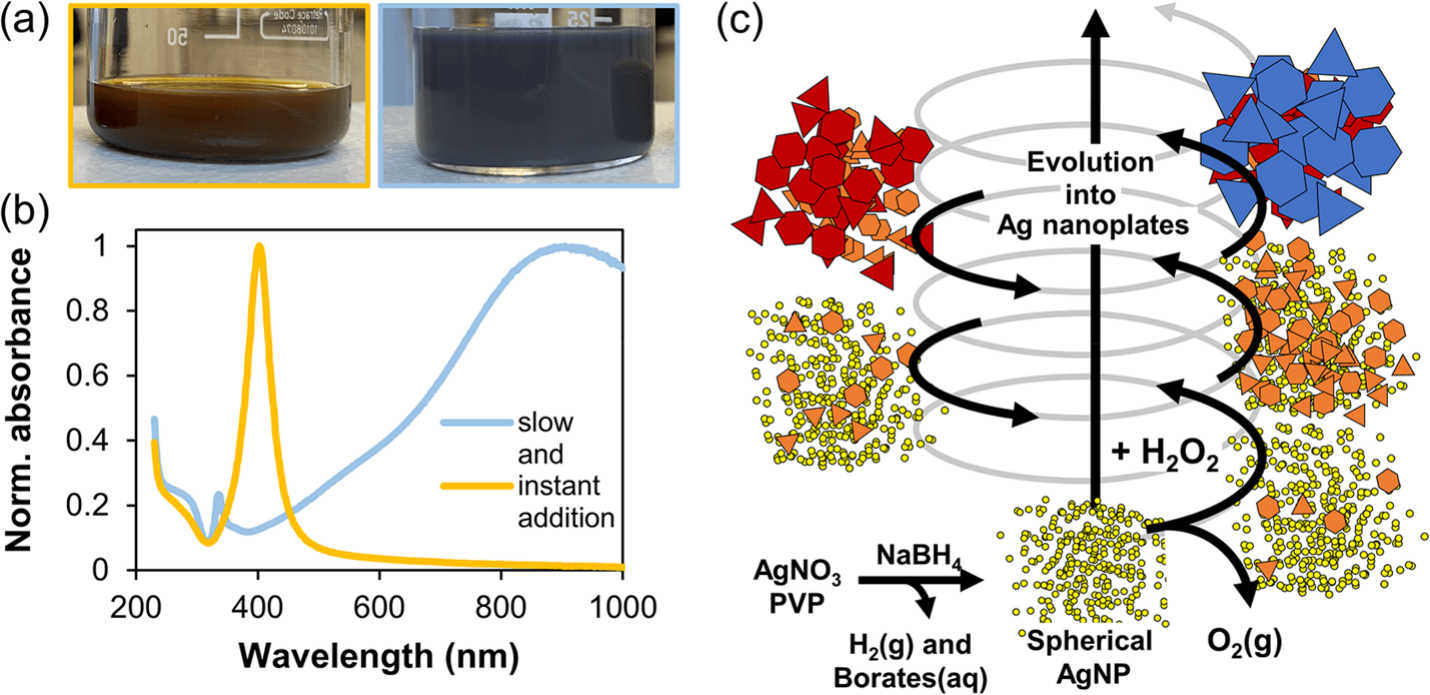
Influence of H_2_O_2_ addition rate on silver nanoplate formation. Photo of the final colloids (a), the corresponding UV–vis absorption spectra for slowly and instantly added H_2_O_2_ (b), and a schematic of the evolution transformation process of spherical AgNP into a nanoplate morphology (c).

Furthermore, this encouraged us to investigated whether in the presence of antifoam an instant addition of the entire amount of H_2_O_2_ would allow synthesis of colloids with the same LSPR maximum as with a gradual addition. For this purpose, we performed two experiments using the same starting solutions and added the same amount (2.2 mL) of 30% H_2_O_2_ at a flow rate of either 0.1 or 100 mL/min. The photos and corresponding UV–vis spectra shown in Figure 5a,b indicate that the mechanism of silver nanoplate growth does not allow immediate AgNP transformation. When H_2_O_2_ is added instantly, it seems that its decomposition is the prevalent reaction and only a minor part contributes to the nanoplate formation. Only when addition is slow and the H_2_O_2_ concentration in the colloid is kept low, efficient transformation based on the oxidation/reduction reaction pathway is observed (Figure 5c).

### Increasing AgNO_3_ and NaBH_4_ Concentration

**Figure 6:**
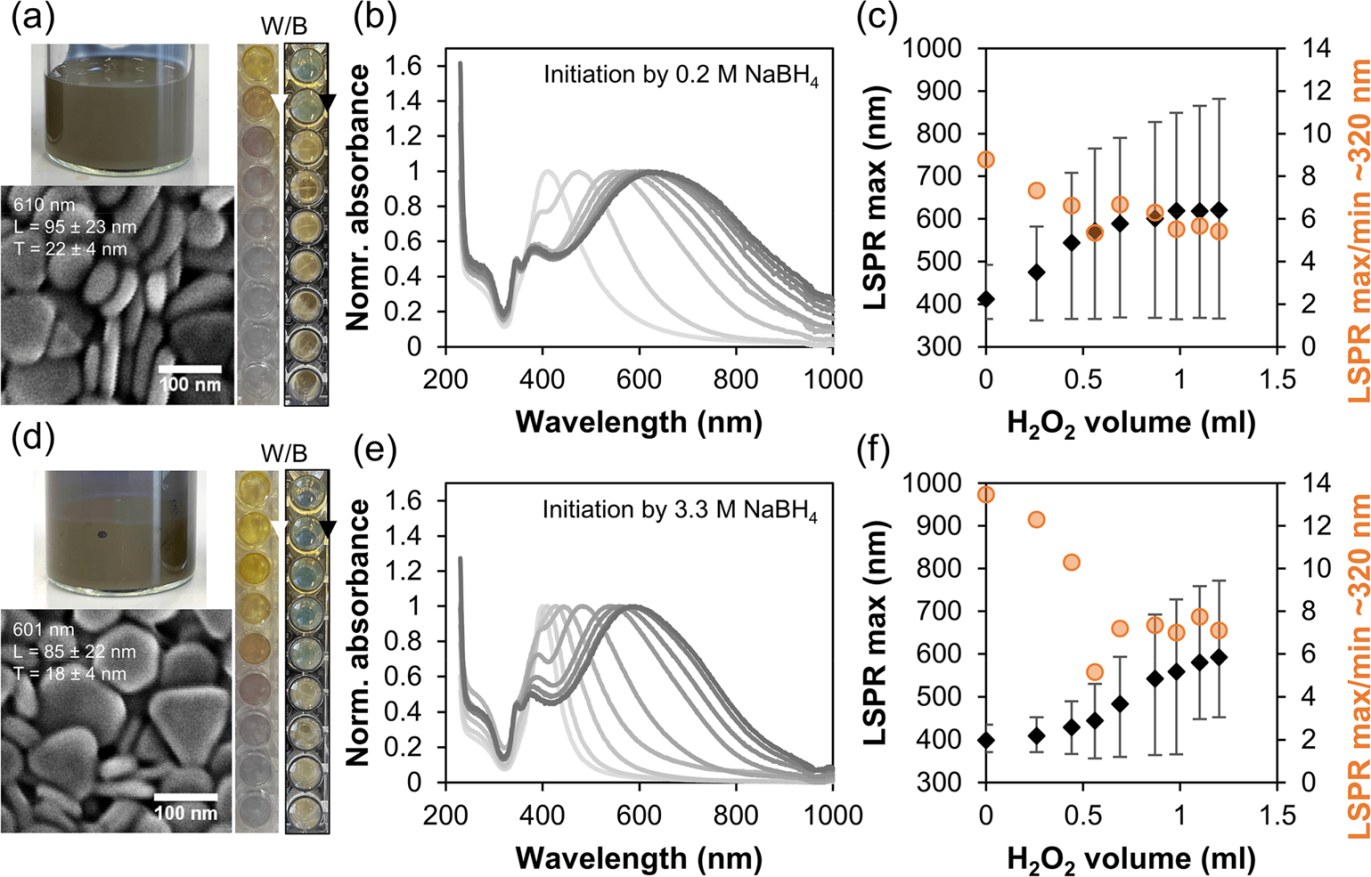
Effect of reagent concentrations on silver nanoplates. Photo of 385-fold diluted samples over white and black background, photo of the final colloid with10-fold increased concentrations, and the corresponding SEM image for the synthesis initiated by 0.2 (a) and 3.3 M NaBH_4_ (d), with corresponding UV–vis spectra (b,e) and their analysis showing the change in LSPR maximum position with fwhm as error bars together with the absorbance ratio of LSPR maximum to minimum at ∼320 nm (c,f).

Results in Figure 4d-f demonstrate that a 5-fold increase in growth solution volume does not affect the quality of the synthesized nanoplates. This was further tested in a volume range between 3 and 450 mL of growth solution, therefore with a volume change of over 2 orders of magnitude. The same results were obtained, demonstrating scalability of the proposed approach (data not shown). In parallel, we investigated whether it is possible to increase the concentration of silver nanoplates while achieving the same quality of colloid coloration. To this end, we decided to increase the concentrations of PVP and AgNO_3_ by 10-fold and add 10-fold the amount of all the other reagents. One should keep in mind that due to a semibatch preparation procedure the concentration of the final product depends on the dilution caused by the initial reduction of Ag^+^ and the subsequent addition of H_2_O_2_. This can be minimized using highly concentrated H_2_O_2_; however, since 30% H_2_O_2_ is the highest concentration available without posing a safety risk, this concentration was kept. Ultimately, the only remaining reagent that can be changed is NaBH_4_. To limit reagent consumption, high concentration experiments were performed in volume of 3 mL growth solution. If the ratio of consumed H_2_O_2_ per Ag^+^ ions remain constant regardless of the reagent concentrations, it can be calculated that when increasing AgNO_3_ concentration by 10-fold, this results in silver nanoplates with a LSPR maximum at 800 nm in concentration increasing from 0.88 g/L to 3.8 g/L, while even 3.3 M NaBH_4_ provides only a moderate further increase in concentration to 4.8 g/L. Furthermore, the results in Figure 6a,d show grayish cloudy colloids indicating thicker crystals later confirmed by SEM images. A comparison between Figure 6b,e shows that when more concentrated NaBH_4_ is used narrower LSPR peaks are obtained as demonstrated by fwhm (error bars) in Figure 6c,f. These results indicate that high concentration reagents can be used for the synthesis of silver nanoplates. Therefore, it is reasonable to assume that in practice, concentrations of at least up to 5 g/L can be prepared, being comparable to the highest reported concentration.^15^

### Characterization of the Prepared Silver Nanoplates

**Figure 7:**
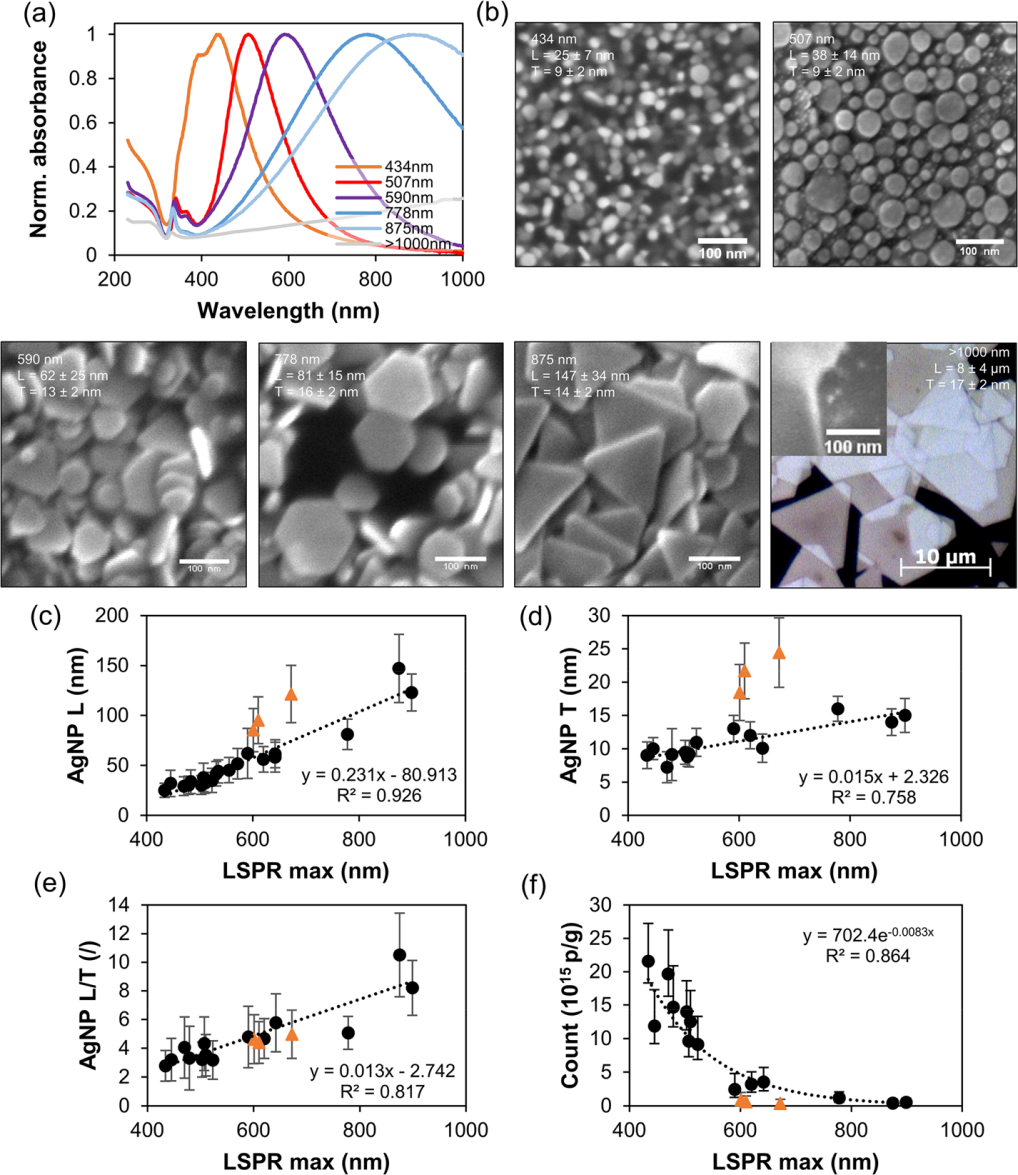
Microscopic analysis of the synthesized and centrifuged silver nanoplates. UV–vis absorbance spectra of the observed colloids (a), with corresponding SEM and optical microscopy images (b); particle length (L), thickness (T), and L/T ratio for prepared silver nanoplates at initial concentration of 1.02 and 10.2 g/L Ag^+^, shown with black points and orange triangles respectively (c-f), and calculated particle number per gram of elemental silver versus LSPR maximum position (f). Measured and calculated standard deviations are shown with error bars (c-f).

Using the developed procedure, different colloids were prepared simply by stopping the addition of H_2_O_2_ at the desired position of the LSPR maximum. Figure 7a shows the spectra obtained after centrifugation and redispersion in DI water for SEM sampling. The typical deep UV–vis absorption of PVP and AgNO_3_ is now absent, indicating their successful removal, while colloids are still stable due to zeta potential of silver.^82^Figure 7b shows the corresponding SEM images of the transformation from preferentially circular nanoplates, exhibiting a low LSPR maximum, to trigonal and hexagonal nanoplates with a higher LSPR maximum. A light microscope image of the large crystals formed after prolonged transformation is shown as well. It is intriguing that even visible light makes the large silver nanoplates appear transparent, confirming their extreme thinness, demonstrated also by the SEM image in the inset. A length to thickness ratio (L/T) of the crystals far exceeded 100, similar to reported values,^47^ indicating that only the hcp arrangement of silver atoms is present.^19^ Despite the large size of the crystals, only the (111) facets are clearly visible, suggesting a large number of stacking faults and twin boundaries.^18,19^ The images show the expected increase in particle length (L) and thickness (T) with LSPR maximum position^33,46^ for which correlations are plotted in Figure 7c-e. Black points represent crystals prepared with Ag^+^ initial concentration of 1.02 g/L, while orange triangles are for the 10-fold concentrated colloid shown in Figure 6a,d. To further elucidate the transformation process, we also estimated the change in nanoparticle number during transformation. Because the SEM images in Figure 7b show that all three morphologies of silver nanoplates are present, we assumed that the particle volume can be approximated with a solid cylinder with a diameter equal to L and a height equal to T. In Figure 7f, the calculated number of silver nanoplates per gram of Ag is plotted against the LSPR maximum, based on data from Figure 7c,d, excluding the orange points. The result demonstrates that the number of particles decreases dramatically during their growth, due to Oswald ripening, namely by 42 times for LSPR maximum change from 450 to 900 nm.

It is important to mention that the centrifugation time depends on the size of the silver nanoplates. In addition, consecutive removal of other components, especially PVP, resulted in faster settling when centrifugation was performed several times. While this seems to be advantageous, it was also observed that the absence of PVP caused stronger adhesion of the nanoplates to surfaces such as glass and polypropylene.

### Drying of the Silver Colloids and Nanoplate Storage

**Figure 8:**
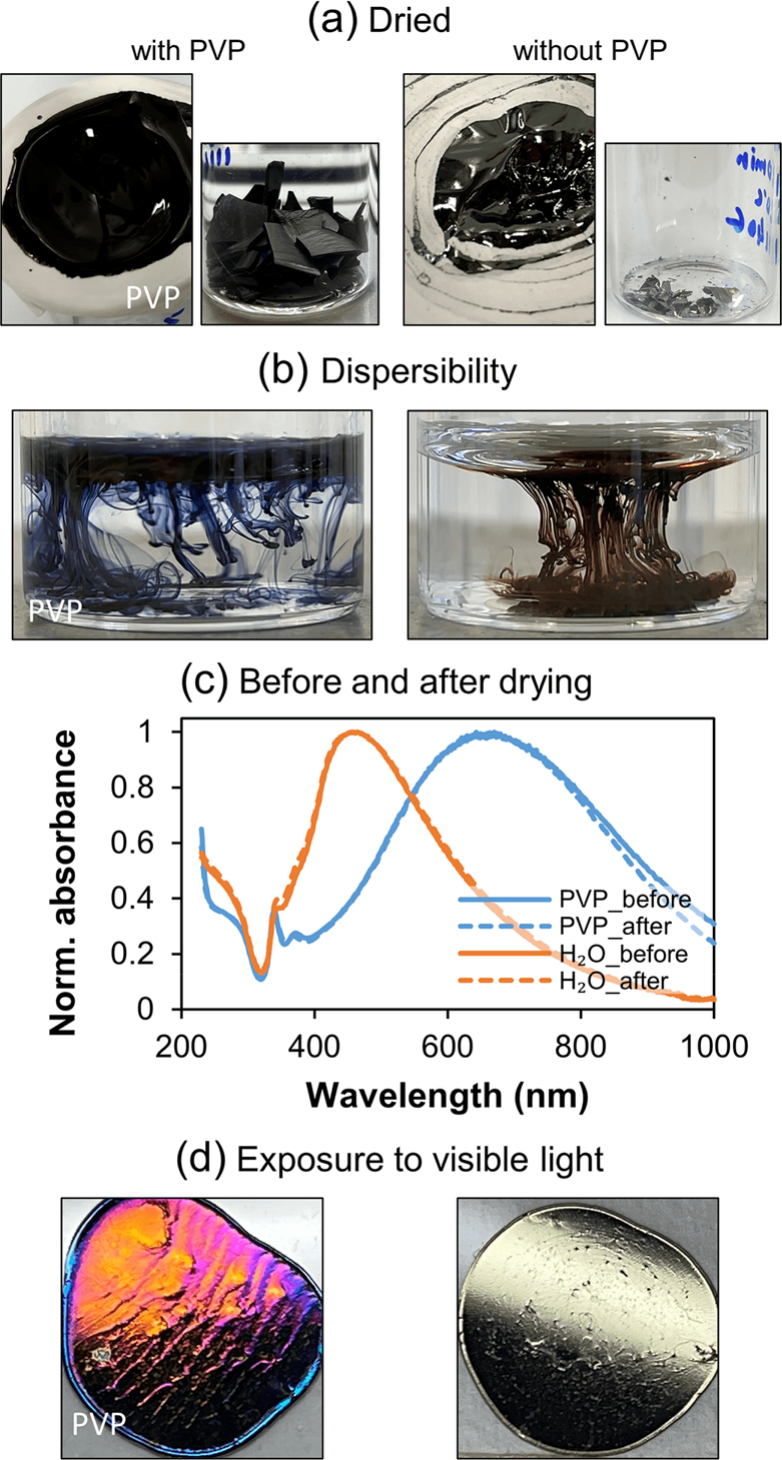
Concentration by drying, dispersibility, storage, and light sensitivity of the colloids. Photograph of dried colloids prepared directly from silver nanoplate colloids with PVP and purified by two centrifugations in H_2_O (a), photo of their dispersal in DI water (b), UV–vis absorbance spectrum comparison of the original solution and solution obtained by redispersing silver nanoplates being dry for one month (c), and dried colloidal droplets after a month of exposure to light at ambient conditions (d).

We showed that Ag nanoplates with concentrations up to almost 5 g/L can be prepared, but even higher concentrations may be required for their transport or special applications, e.g., pigment production for acrylics. To further increase the concentration, centrifugation can be used as discussed previously, and much higher concentrations can be obtained. When the entire liquid is to be removed or we lack the appropriate equipment, evaporation can be used instead. Figure 8a shows photos of the colloids dried at 60 °C in the dark on PTFE tape with and without PVP (removed by two consecutive centrifugations and redispersion in DI water). Both samples were stored in the dark for one month and redispersed in DI water as shown in Figure 8b, afterward. After resuspension was completed, no remaining agglomerates were detected, indicating a high stability of silver nanoplates when dry. The main difference was only in resuspension time, which took longer for samples without PVP, as seen by comparing Videos S1 and S2 (Supporting Information). In Figure 8c the corresponding normalized UV–vis absorbance spectrum of the original and dried colloids dispersed in DI water is shown. No significant changes in the LSPR maximum are present, confirming complete redispersion of dried silver nanoplates. Close spectra overlap also indicates that no oxidation took place, which is easily detected by a red shift in the LSPR maximum.^83^ Finally, stability of high concentration dried droplets^84^ of silver nanoplates with and without PVP was tested toward light at ambient conditions. In Figure 8d the photos of both samples exposed to natural light for 1 month are shown. While the sample without PVP preserved original color, the one with PVP become intensively colored, indicating probable secondary growth of anisotropic silver nanoparticles. To our best knowledge this is the first observation of silver nanoplate solid phase growth, opening potential novel applications of silver nanoplates, such as detection systems for light exposure.

This study resulted in a method that allows daily preparation of gram quantities of silver nanoplates with the targeted LSPR maximum even with a growth solution volume of less than 1 L. Such colloids can be completely dried, either with the remaining PVP or when removed, exhibiting long-term stability when stored in the dark. Resuspension of the dried silver nanoplates in DI water is straightforward and takes only a few minutes, even without mixing (see Videos S1 and S2). This simplifies their use in various applications and opens the possibility for their wide implementation.

### Application of Ag Nanoplates as Pattern Formation Pigment in Modern Art

**Figure 9:**
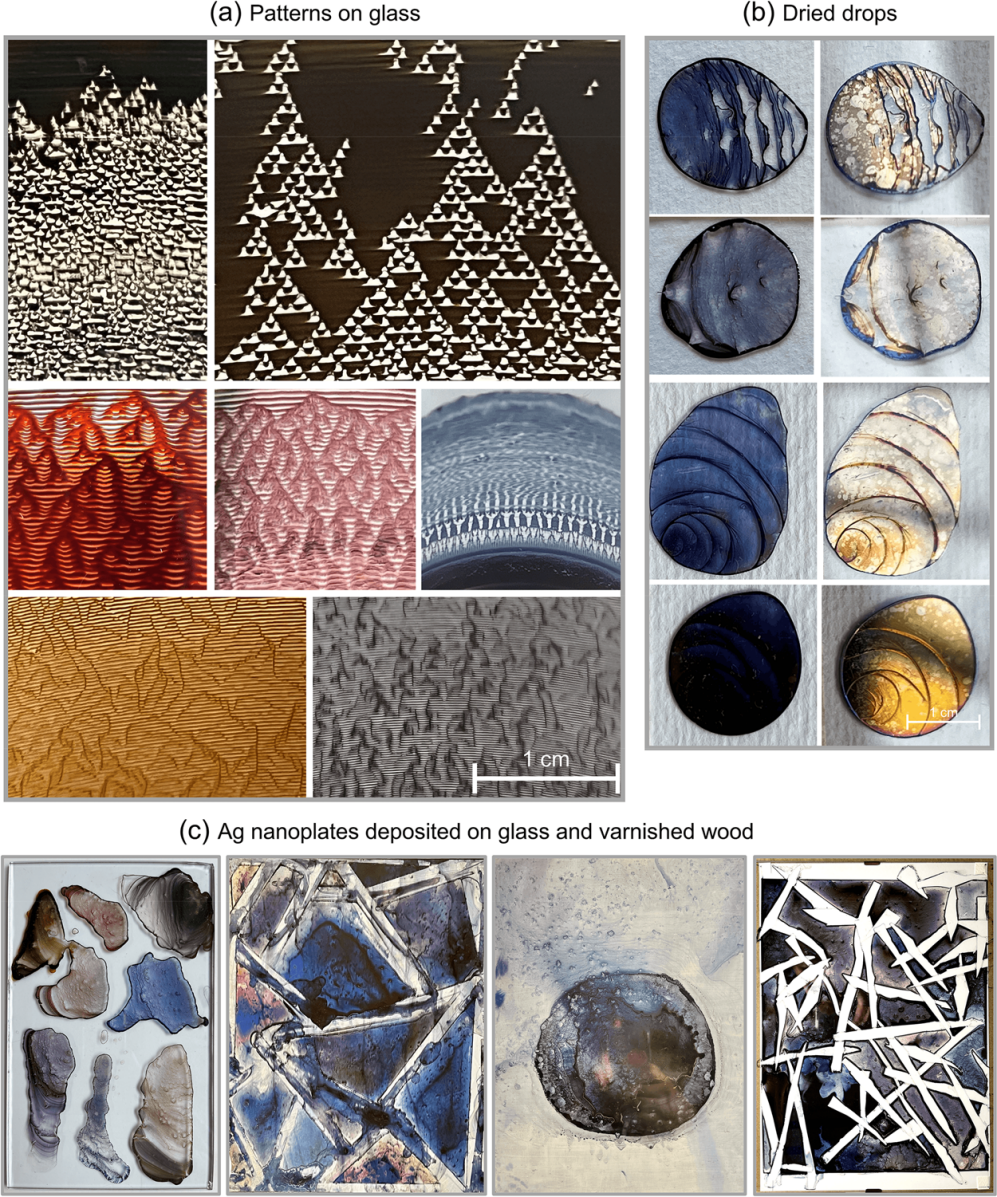
Application of silver nanoplates. Patterns of deposited Ag nanoplates after drying on glass immersed in beakers filled with colloid (a); pattern formation after drying drops of Ag nanoplate colloids with PVP showing secondary nucleation on images with reflected light (b); amateur art on glass and varnished wood with Ag nanoplates (c).

The ease of silver nanoplate preparation in large quantities, the possibility of their storage and therefore facilitated supply, further increases their applicability. While many applications are discussed in detail in the literature (see Introduction section), another potential application is in the modern art^11,85^ where large quantities are needed at relatively low cost. The colloids are composed of uniform, highly asymmetric particles and can not only express different colors but also, when combined with soluble polymer and salt after drying, produce a variety of patterns based on self-pinning.^86^ In Figure 9, a variety of different patterns, ranging from rather simple multi-coffee-ring shapes^87^ to dendrite-like structures and even more complex fractal patterns. These phenomena, combined with colors that cover the entire visible spectrum and beyond, make silver-based plasmonic materials a unique pigment for use in art. Figure 9a shows a macroscopic pattern of conical structures previously observed in dried poly(ethylene oxide) droplets.^88^ Under appropriate drying conditions, they develop fractal patterns, such as multilevel Sierpiński triangles, recently described at the molecular level,^89,90^ that also resemble the patterns formed by polystyrene microspheres stabilized with sodium dodecyl sulfate (SDS).^86^ In Figure 9b the coffee-ring effect,^91^ the multiring pattern due to stick–slip motion,^86^ and a nearly uniform deposition^86^ are presented. This pattern formation is affected by particle concentration and additives,^86^ temperature,^87^ and particle shape.^92^ In addition, a reflected light image shows the metallic nature of the silver nanoplates when deposited on flat surfaces, forming a mirror with clearly visible secondary yellow AgNP nuclei.

As silver nanoparticles have already found their way into art,^93^ we decided to also show our amateur abstract paintings with silver nanoplates in Figure 9c. It is worth noting that while these colors look amazing in person, they are difficult to capture in a photo because of reflections. However, the combined result shows that the use of silver nanoplates as a new pigment holds great potential for unprecedented artistic expression. Needless to say, such application is not limited to art, but can also be extended to preparation of plasmonic textiles, expressing also antibacterial and fungicidal properties.^94,95^

## Conclusion

Silver nanomaterials are promising plasmonic materials with myriad existing and emerging applications. Silver nanoplates, which enable plasmonic resonance across the entire visible spectrum, are certainly one of the most promising morphologies and are becoming increasingly popular with the advance of fast light-based technologies, increasing the demand for their efficient fabrication. The developed method can be easily applied when large quantities of silver nanoplates with precise optical properties are needed. Due to its simplicity, scalability, high productivity, and reproducibility of the targeted plasmonic properties, its transfer to industrial scale seems to be straightforward, even for production under good manufacturing practice (GMP), which is required for products used on humans. Furthermore, their handling and reuse is very easy due to their demonstrated long-term stability in dry form, allowing better accessibility of silver nanoplates. Because of that, they can be expected to become a drawing card in applications where target plasmonic properties at optical frequencies are required, including art.

## Supporting Information Available

Video S1: dissolution of Ag nanoplates in DI water (MP4)
Video S2: dissolution of Ag nanoplates in DI water (MP4)
